# Combined Association of the Fibrinogen-to-Albumin Ratio and the Uric Acid-to-Albumin Ratio with Mortality in Critically Ill Patients with Acute Kidney Injury Receiving Continuous Renal Replacement Therapy: A Retrospective Cohort Study

**DOI:** 10.3390/jcm15093271

**Published:** 2026-04-24

**Authors:** Jun Shang, Li Wei, Shiyu Chen, Xuemin Tang, Yitong Zhu, Xunliang Li, Ruifeng Wang

**Affiliations:** 1 Department of Nephrology, The Second Affiliated Hospital of Anhui Medical University, Hefei 230000, China; junshang@stu.ahmu.edu.cn (J.S.); 2445011933@stu.ahmu.edu.cn (L.W.); 2545012075@stu.ahmu.edu.cn (S.C.); 2436020088@stu.ahmu.edu.cn (X.T.); 2 Department of Nephrology, Anhui Medical University, Hefei 230000, China; 3 Institute of Kidney Disease, Inflammation & Immunity Mediated Diseases, Anhui Medical University, Hefei 230000, China; 4 Department of Nephrology, Wuhu No. 1 People’s Hospital, Wuhu 241000, China; 5 Division of Life Sciences and Medicine, University of Science and Technology of China, Hefei 230000, China; zhuyitong1999@mail.ustc.edu.cn

**Keywords:** acute kidney injury, continuous renal replacement therapy, fibrinogen-to-albumin ratio, uric acid-to-albumin ratio, mortality, retrospective cohort study, MIMIC-IV

## Abstract

**Background:** The combined prognostic value of the fibrinogen-to-albumin ratio (FAR) and uric acid-to-albumin ratio (UAR) in acute kidney injury patients undergoing continuous renal replacement therapy remains unclear. **Methods:** This retrospective cohort study utilized the MIMIC-IV database. Adult patients with AKI receiving CRRT were included and stratified into four groups based on optimal FAR and UAR cut-offs. Multivariable Cox proportional hazards regression and restricted cubic spline analyses were employed to examine associations with 30-, 90-, and 360-day all-cause mortality. **Results:** Patients with high FAR/high UAR had the poorest survival (log-rank *p* < 0.001). After multivariable adjustment, high FAR/high UAR was associated with higher 30-day (HR = 2.17, 95%CI: 1.61–2.92) and 360-day mortality (HR = 1.50, 95%CI: 1.18–1.90) vs. low FAR/low UAR. The association was stronger in patients with an SOFA score > 12 or vasopressin use (interaction *p* < 0.05). **Conclusions:** In critically ill AKI patients undergoing CRRT, the combined assessment of the FAR and UAR is associated with elevated mortality risk. These readily obtainable composite markers may support risk stratification in clinical practice.

## 1. Introduction

Acute kidney injury (AKI) is a clinical syndrome characterized by a rapid decline in renal function, leading to the accumulation of nitrogenous waste products, electrolyte imbalances, and fluid disturbances. It is associated with multiple organ dysfunction and, in some cases, even death [1]. National, regional, and global statistics indicate that AKI affects one in five hospitalized adults and 50% to 60% of critically ill patients [2]. Due to differences in healthcare resources, diagnostic criteria, and disease spectrum across countries and regions, the epidemiological characteristics of AKI exhibit certain heterogeneity. Nevertheless, overall, AKI significantly prolongs hospital stays, increases healthcare burden, and leads to extremely high short-term and long-term mortality [3]. For patients with severe AKI unresponsive to pharmacological therapy, continuous renal replacement therapy (CRRT) has become a crucial life-support measure. CRRT achieves better hemodynamic stability and fluid balance through the continuous, gradual removal of solutes and water, making it particularly suitable for critically ill patients [4,5]. However, despite the increasing adoption of CRRT technology, mortality rates among AKI patients receiving this treatment remain strikingly high, ranging between 50% and 70% [6,7]. This grim reality suggests that relying solely on CRRT support is insufficient to significantly improve patient survival outcomes, underscoring an urgent need to establish effective prognostic stratification models to guide early risk stratification, individualized treatment decisions, and resource allocation.

Among the various factors influencing AKI prognosis, nutritional and inflammatory status has garnered increasing attention. As a negative acute-phase protein, serum albumin is a classic indicator reflecting both nutritional status and the level of systemic inflammation, with hypoalbuminemia being closely associated with poor outcomes in critically ill patients [8,9,10,11]. Concurrently, fibrinogen, as an acute-phase protein, increases significantly during states of inflammation [12]. Recent research suggests that the fibrinogen-to-albumin ratio (FAR), a novel composite marker of inflammation and nutrition, has been linked to the occurrence of septic acute kidney injury and in-hospital mortality in critically ill patients [13]. On the other hand, elevated uric acid levels are often associated with conditions such as hypertension, diabetes, and chronic kidney disease. Furthermore, hyperuricemia itself is an independent risk factor for both the development and progression of AKI, and it is associated with increased mortality risk [14]. Recent studies have proposed that the uric acid-to-albumin ratio (UAR), an easily accessible composite index, is associated with 28-day mortality in AKI patients [15,16].

Although the fibrinogen-to-albumin ratio and the uric acid-to-albumin ratio have each shown prognostic relevance in patients with acute kidney injury, the rationale for evaluating them jointly is particularly important in critically ill patients receiving continuous renal replacement therapy. The fibrinogen-to-albumin ratio integrates two biologically complementary signals: elevated fibrinogen as a marker of acute-phase inflammatory activation and reduced albumin as an indicator of impaired nutritional reserve and systemic inflammatory burden [13]. In parallel, the uric acid-to-albumin ratio may capture metabolic and renal dysfunction in a context that is also influenced by hypoalbuminemia, which itself reflects illness severity and inflammatory stress [15]. Thus, these two ratio-based biomarkers may represent related but non-identical pathophysiological domains, including inflammation, nutritional impairment, and metabolic derangement. Because patients with acute kidney injury receiving continuous renal replacement therapy commonly present with profound systemic inflammation, hemodynamic instability, and multisystem dysfunction [5], a combined evaluation of these two biomarkers may offer a more clinically relevant framework for risk stratification than either marker alone. However, this combined association has not been systematically evaluated in this specific high-risk population.

Accordingly, in this retrospective cohort study using the MIMIC-IV database, our primary objective was to examine the association between combined fibrinogen-to-albumin ratio and uric acid-to-albumin ratio categories and 30-day all-cause mortality in critically ill patients with acute kidney injury receiving continuous renal replacement therapy. Our secondary objectives were to assess the associations of these biomarker categories with 90-day and 360-day all-cause mortality, to characterize the dose–response relationships of the fibrinogen-to-albumin ratio and uric acid-to-albumin ratio with 30-day mortality, and to explore whether these associations differed across clinically relevant subgroups.

## 2. Materials and Methods

### 2.1. Study Design and Data Source

This was a retrospective cohort study conducted in accordance with the Strengthening the Reporting of Observational Studies in Epidemiology (STROBE) statement [17]. The completed STROBE checklist is provided as Supplementary Materials. The study utilized data from the Medical Information Mart for Intensive Care-IV (MIMIC-IV, v3.1) database [18]. This publicly available database contains detailed, de-identified clinical data of critically ill patients admitted to the Beth Israel Deaconess Medical Center in Boston, MA, USA, between 2008 and 2019. The use of this database was approved by the Institutional Review Board of the Massachusetts Institute of Technology (requirement for informed consent was waived due to the de-identified nature of the data). To ensure patient privacy and standardized data use, all researchers completed the online course “Protecting Human Research Participants” and passed the examination before obtaining access to the database (certification ID: 62150647).

### 2.2. Participants

**Figure 1:**
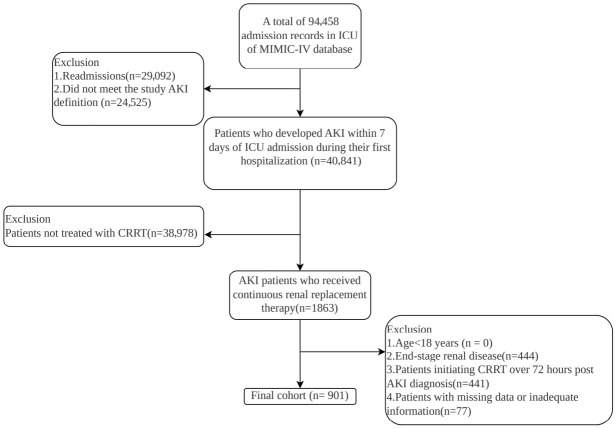
Flow diagram of patient selection. From an initial set of 94,458 ICU admission records in the MIMIC-IV database, 901 patients with acute kidney injury (AKI) who received continuous renal replacement therapy (CRRT) and met all inclusion criteria were included in the final cohort.

All adult patients (≥18 years) from the MIMIC-IV database with a first admission to the intensive care unit (ICU) were screened for eligibility. The inclusion criteria were as follows: (1) age ≥ 18 years; (2) diagnosis of AKI during the first ICU admission, defined according to the Kidney Disease: Improving Global Outcomes (KDIGO) criteria [19]; and (3) receipt of CRRT following AKI diagnosis. The exclusion criteria were as follows: (1) pre-existing end-stage renal disease (ESRD); (2) initiation of CRRT more than 72 h after AKI diagnosis; and (3) missing data for any key laboratory values required to calculate the FAR (fibrinogen, albumin) or UAR (uric acid, albumin). The patient selection process is illustrated in detail in Figure 1.

### 2.3. Variables

The primary exposure variables were the FAR and the UAR, calculated using the first recorded fibrinogen, uric acid, and albumin measurements obtained after ICU admission. The primary outcome was 30-day all-cause mortality after ICU admission. Secondary outcomes were 90-day and 360-day all-cause mortality. Patients were followed from ICU admission until death or the end of the corresponding follow-up period, whichever occurred first.

Potential covariates included demographic characteristics (age, sex, race, and body mass index [BMI]); disease severity scores (Acute Physiology Score III [APS III] and Sequential Organ Failure Assessment [SOFA] score) calculated within the first 24 h of ICU admission; laboratory variables measured after ICU admission; critical care interventions, including mechanical ventilation and vasoactive drug use; and comorbidities identified using ICD-9/ICD-10 codes. A complete list of baseline variables is provided in Table 1.

### 2.4. Data Sources and Measurement

All data were extracted from the MIMIC-IV database using Structured Query Language (SQL). When multiple laboratory values were available, the first recorded value after ICU admission was used for exposure assessment and baseline laboratory covariates. APS III and SOFA scores were derived from physiological data collected during the first 24 h of ICU stay. Comorbidities were identified from diagnostic records using ICD-9 and ICD-10 codes documented in the database.

### 2.5. Bias and Missing Data

Several steps were taken to improve internal validity. To reduce selection heterogeneity, the cohort was restricted to adult patients with a first ICU admission, AKI defined according to KDIGO criteria, and CRRT initiated after AKI diagnosis. To reduce temporal heterogeneity in exposure assessment, FAR and UAR were calculated using the initial laboratory measurements after ICU admission. To address potential confounding, multivariable Cox proportional hazards models were adjusted for clinically relevant covariates selected using a combination of clinical judgment and statistical selection procedures. Patients with missing laboratory data required to calculate FAR or UAR were excluded during cohort selection. For multivariable analyses, a complete-case approach was used. In addition, because severe hepatic dysfunction may influence both fibrinogen and albumin levels, a sensitivity analysis excluding patients with cirrhosis was performed to assess the robustness of the observed association between FAR and mortality.

### 2.6. Study Size

No a priori sample size calculation was performed because this study included all eligible patients available in the MIMIC-IV database during the study period. The final study cohort consisted of 901 patients.

### 2.7. Quantitative Variables

Continuous variables were assessed for approximate normality by visual inspection of histograms and Q-Q plots. Normally distributed continuous variables are presented as mean ± standard deviation, whereas non-normally distributed continuous variables are presented as median and interquartile range. For survival analyses, optimal cut-off values for FAR and UAR were determined using receiver operating characteristic (ROC) curve analysis based on the Youden index, and patients were categorized into four combined exposure groups accordingly. In secondary analyses, FAR and UAR were also modeled as continuous variables using restricted cubic splines (RCS) to examine dose–response relationships.

### 2.8. Statistical Analysis

Baseline characteristics were compared between 30-day survivors and non-survivors. Categorical variables are presented as frequencies (percentages) and were compared using the Chi-square test or Fisher’s exact test, as appropriate. Continuous variables were compared using the independent-samples *t* test or Mann–Whitney U test, as appropriate.

Restricted cubic spline analyses within Cox proportional hazards models were used to assess dose–response relationships between continuous FAR/UAR values and 30-day mortality. Kaplan–Meier survival curves were generated for the four combined FAR/UAR groups, and differences in survival were compared using the log-rank test.

Cox proportional hazards regression models were constructed to estimate the association between combined FAR/UAR groups and mortality. Model 1 was unadjusted. Model 2 was adjusted for age, sex, and BMI. For Model 3, candidate covariates were selected from clinically relevant baseline variables using a combination of clinical judgment and forward stepwise selection (entry *p* < 0.1), with age, sex, and BMI forced into the model. The final adjusted model included sepsis, diabetes, acute myocardial infarction, vasopressin use, SOFA score, red cell distribution width, neutrophil count, partial pressure of oxygen, serum creatinine, serum magnesium, and anion gap. The proportional hazards assumption was assessed using scaled Schoenfeld residuals.

Pre-specified subgroup analyses were performed according to age, sex, BMI, sepsis, diabetes, SOFA score, mechanical ventilation, and vasopressin use. Interaction terms were tested in the fully adjusted model. All statistical analyses were performed using R software version 4.0.3 (R Foundation for Statistical Computing, Vienna, Austria), and a two-sided *p* value < 0.05 was considered statistically significant.

## 3. Results

### 3.1. Baseline Characteristics of the Study Population

A total of 901 AKI patients receiving CRRT were ultimately included in the study. Based on 30-day survival status, patients were divided into a survival group (*n* = 433) and a non-survival group (*n* = 468). The baseline characteristics of the two groups are shown in Table 1. Compared with the non-survival group, survivors were younger (median age: 61 vs. 65 years, *p* < 0.01). Regarding illness severity, the non-survival group had significantly higher APS III scores (88 vs. 76, *p* < 0.01) and SOFA scores (12 vs. 11, *p* < 0.01), suggesting worse baseline condition and more severe organ failure. Key laboratory indicators showed that the non-survival group had a significantly higher FAR (6.67 vs. 5.52, *p* < 0.01) and UAR (1.77 vs. 1.50, *p* < 0.01). Furthermore, the non-survival group had higher levels of fibrinogen, uric acid, white blood cell count, neutrophil count, and anion gap but lower albumin levels and PO2. In terms of treatments, the non-survival group had a higher usage of norepinephrine (61.97% vs. 49.42%, *p* < 0.01), epinephrine (20.30% vs. 12.93%, *p* < 0.01), and vasopressin (41.67% vs. 27.71%, *p* < 0.01). Regarding comorbidities, the non-survival group had a higher incidence of sepsis (62.61% vs. 50.35%, *p* < 0.01) and acute myocardial infarction (19.44% vs. 12.70%, *p* < 0.01), while diabetes was more common in the survival group (36.95% vs. 29.70%, *p* = 0.02).

### 3.2. Determination of FAR and UAR Cut-Off Values

ROC curve analysis was used to evaluate the discriminative ability of the FAR and UAR for 30-day mortality risk (Supplementary Figure S1). The results indicated that both had good discriminative ability for the short-term prognosis of AKI-CRRT patients. The optimal cut-off values for the FAR and UAR, determined using the Youden index, were 5.928 and 1.218, respectively. Accordingly, patients were divided into a high-FAR group (FAR > 5.928) and a low-FAR group (FAR ≤ 5.928), as well as a high-UAR group (UAR > 1.218) and a low-UAR group (UAR ≤ 1.218).

### 3.3. Survival Analysis Based on FAR and UAR Groups

**Figure 2:**
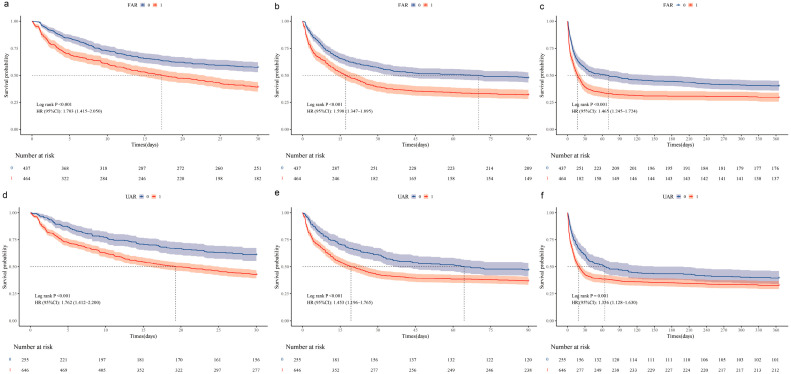
Kaplan–Meier survival curves comparing 30-day (**a**,**d**), 90-day (**b**,**e**), and 360-day (**c**,**f**) all-cause mortality between groups stratified by the fibrinogen-to-albumin ratio (FAR) and the uric acid-to-albumin ratio (UAR). Optimal cut-off values were determined by the Youden index: FAR = 5.928 (high FAR: >5.928; low FAR: ≤5.928); UAR = 1.218 (high UAR: >1.218; low UAR: ≤1.218). Shaded areas represent 95% confidence intervals. The number of patients at risk at each time point is displayed below the x-axis. *p*-values were calculated using the log-rank test.

**Figure 3:**
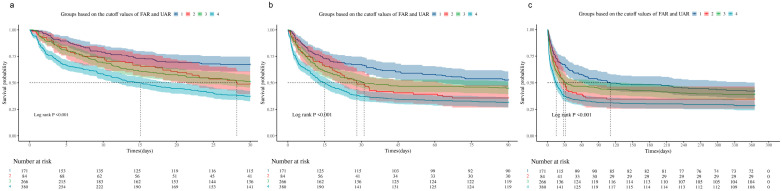
Kaplan–Meier survival curves for 30-day (**a**), 90-day (**b**), and 360-day (**c**) mortality stratified by combined FAR and UAR categories. Patients were divided into four groups: Group 1, low FAR/low UAR; Group 2, high FAR/low UAR; Group 3, low FAR/high UAR; and Group 4, high FAR/high UAR.

Kaplan–Meier survival analysis based on the above groupings showed that during the 30-, 90-, and 360-day follow-up periods, both the high-FAR group (Figure 2a–c) and the high-UAR group (Figure 2d–f) had significantly lower cumulative survival rates compared with their corresponding low-ratio groups (log-rank *p* < 0.05). To further investigate the combined effect of the FAR and UAR, the patients were divided into four groups: Group 1 (low FAR/low UAR), Group 2 (high FAR/low UAR), Group 3 (low FAR/high UAR), and Group 4 (high FAR/high UAR). The survival curves (Figure 3) clearly show that, at all time points (30, 90, and 360 days), Group 4 had the lowest survival probability, while Group 1 had the highest, with statistically significant differences between the groups (log-rank *p* < 0.001).

### 3.4. Dose–Response Relationship Between FAR, UAR, and Mortality Risk

**Figure 4:**
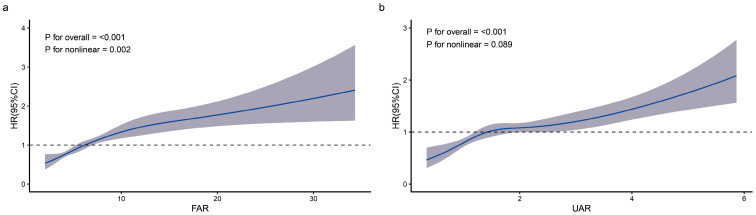
Restricted cubic spline (RCS) analysis of the dose–response relationship between (**a**) the fibrinogen-to-albumin ratio (FAR) and (**b**) the uric acid-to-albumin ratio (UAR) with 30-day all-cause mortality. Solid lines represent the estimated hazard ratio (HR), and shaded bands indicate 95% confidence intervals. The reference values (HR = 1.0) were set at the medians of FAR and UAR.

RCS analysis was employed to examine the potential non-linear associations of the FAR and UAR with 30-day all-cause mortality (Figure 4). A significant non-linear association was observed between the FAR and mortality risk (overall *p* < 0.001, non-linear *p* = 0.002), suggesting that the relationship was non-linear across the range of FAR values. For the UAR, the analysis also suggested that there was a non-linear pattern with mortality risk (overall *p* < 0.001); however, the non-linearity itself was of borderline statistical significance (non-linear *p* = 0.089).

### 3.5. Multivariable Cox Regression Analyses

Univariable and multivariable Cox regression analyses further confirmed the above findings (Table 2). Using Group 1 (low FAR/low UAR) as the reference, the fully adjusted multivariable model (Model 3) showed that, for 30-day mortality, the hazard ratios (HRs) for Group 2 (high FAR/low UAR), Group 3 (low FAR/high UAR), and Group 4 (high FAR/high UAR) were 1.59, 1.61, and 2.17, respectively, which are all statistically significant values. For 360-day mortality, after multivariable adjustment, Group 4 (high FAR/high UAR) showed an independent prognostic association (HR = 1.50, 95%CI: 1.18–1.90, *p* < 0.01), indicating that the combination of both ratios holds significant prognostic relevance for long-term outcomes.

### 3.6. Subgroup Analysis

**Figure 5:**
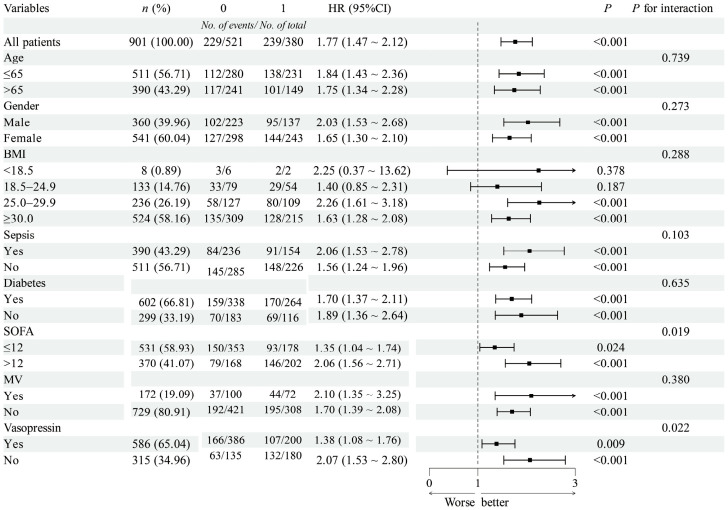
Subgroup analysis of the association between high FAR/high UAR (Group 4) and 30-day mortality. Hazard ratios (HRs) with 95% confidence intervals (CIs) were derived from multivariable Cox models adjusted for age, sex, BMI, sepsis, diabetes, AMI, vasopressin use, SOFA score, RDW, neutrophil count, PO2, creatinine, magnesium, and anion gap. *p*-values for interaction test whether the association differs across subgroups. Abbreviations: BMI, body mass index; SOFA, sequential organ failure assessment; MV, mechanical ventilation.

Subgroup analysis was conducted to explore the association between high FAR/high UAR and 30-day mortality across different patient characteristics (Figure 5). The results showed that the association between high FAR/high UAR and increased mortality risk was consistent across most of the subgroups. However, significant interaction effects were observed for SOFA score (interaction *p* = 0.019) and vasopressin use (interaction *p* = 0.022). Specifically, the magnitude of the association between high FAR/high UAR and mortality risk was significantly greater in patients with SOFA score > 12 (HR = 2.06) and in patients receiving vasopressin (HR = 2.07).

## 4. Discussion

Based on the large critical care database MIMIC-IV, the present study showed that, among patients with AKI receiving CRRT, the coexistence of a high FAR and a high UAR was associated with increased risks of 30-day and 360-day all-cause mortality. In multivariable Cox regression analyses, patients with both high FAR and high UAR remained at significantly increased risk of short-term and long-term death after adjustment for multiple confounders, and these findings were further supported by Kaplan–Meier survival analyses.

The results of this study showed that both a high UAR and a high FAR were significantly associated with mortality risk in patients with AKI, and the association was stronger when the two were combined. The prognostic relevance of uric acid in critical illness has been increasingly recognized; a recent ancillary analysis of the prospective FROG-ICU cohort (URIC-ICU) demonstrated that higher admission serum uric acid levels were independently associated with increased 90-day mortality (HR = 1.43, 95%CI 1.11–1.82) [20]. Regarding UAR, Özgür et al. found, using multivariable Cox regression in patients with AKI, that UAR was independently associated with 30-day mortality (HR = 1.39) [16]. Similarly, Sargin Ertan et al. identified a clear association between UAR and 28-day mortality in critically ill patients [15]. The findings of these two studies are consistent with the conclusions of the present study. Regarding FAR, Zhan et al. reported that in critically ill patients with septic acute kidney injury, a high FAR was significantly associated with an increased risk of 30-day mortality (HR = 1.22) [13], which is in line with our results. Furthermore, a recent retrospective study utilizing the MIMIC-IV database demonstrated that a higher FAR was independently associated with increased all-cause mortality in critically ill adult patients with atrial fibrillation [21], thereby extending the adverse prognostic implication of FAR to a broader spectrum of adult critical illness. In the present study, the 30-day hazard ratio for the high-FAR/low-UAR group was 1.59. Notably, patients with both high FAR and high UAR had the highest HR, suggesting that the combined assessment of the two markers may provide complementary prognostic information. Taken together, these studies support the prognostic relevance of UAR and FAR in AKI or critically ill populations. On this basis, to our knowledge, the present study is among the first to examine the combined association of these two ratios in patients with AKI receiving CRRT.

However, not all studies support the association between the above ratios and mortality risk. Chen et al., based on the MIMIC-III database, found that serum uric acid alone was not significantly associated with 90-day mortality in ICU patients (HR = 1.00) [22]. In contrast, in our study, the combination of uric acid and albumin showed a significant association (30-day HR = 2.17 for the high-FAR/high-UAR group), suggesting that uric acid used alone is susceptible to confounding factors. He et al. reported that in critically ill pediatric patients, a high FAR was associated with a decreased risk of mortality (HR = 0.83) [23], which is opposite to our finding that the high-FAR/low-UAR group had a 30-day mortality HR of 1.59. This discrepancy may be explained by differences in the inflammatory compensatory mechanisms between children and adults. Moreover, all patients in our study were receiving CRRT for AKI and were more severely ill; in this context, a high FAR reflects a hypercoagulable state and poor outcomes.

From a biological mechanism perspective, the association between elevated FAR and UAR and poor prognosis in patients with CRRT-AKI primarily involves the loss of albumin function and the pathological effects of fibrinogen and uric acid. Albumin not only serves as the main maintainer of plasma colloid osmotic pressure but also plays an important antioxidant role by scavenging reactive oxygen and nitrogen species through its free thiol group at Cys34, while also exhibiting esterase activity and anti-inflammatory properties [24]. In the context of AKI and CRRT treatment, patients are often in a state of systemic inflammation and oxidative stress, in which albumin synthesis is suppressed and oxidative modification is increased, leading to a decline in its antioxidant and endothelium-protective functions. Amouzandeh et al. found in patients with advanced chronic liver disease that the absolute synthesis rate of albumin was significantly negatively correlated with Child–Pugh score and MELD score, and that low albumin synthesis coexisted with an inflammatory state [25]. This explains why, when albumin levels are low, UAR is associated with an increased risk of mortality: low albumin impairs the body’s ability to scavenge reactive oxygen species and stabilize the endothelial glycocalyx, while elevated uric acid further promotes endothelial dysfunction and inflammatory responses by inducing NADPH oxidase activation [24], thereby creating a vicious cycle of “low antioxidant capacity–high oxidative stress”. Regarding FAR, fibrinogen, as an acute-phase reactant, has its synthesis significantly upregulated under inflammatory conditions. Amouzandeh et al. found that although albumin synthesis is reduced in patients with liver failure, fibrinogen synthesis can remain normal or even increase due to chronic inflammation, and is positively correlated with CRP levels [25]. High fibrinogen promotes platelet aggregation, increases plasma viscosity, and impairs endothelial integrity, thereby elevating the risk of thrombosis. Meanwhile, the decrease in colloid osmotic pressure and tissue edema caused by low albumin can further exacerbate microcirculatory disturbances. Thus, an elevated FAR reflects a dual hit of procoagulant and anti-inflammatory/antioxidant imbalance. In patients with AKI receiving CRRT, the extracorporeal circulation itself can activate inflammatory and coagulation cascades. As integrated indicators, FAR and UAR can comprehensively reflect the patient’s inflammatory–oxidative–coagulation network status, thereby providing a more complete assessment of mortality risk than either marker alone.

The present study found that FAR exhibited a significant nonlinear association with 30-day mortality (*p* for nonlinearity = 0.002), whereas the nonlinearity for UAR was only borderline significant (*p* = 0.089). In contrast to the inverted N-shaped relationship reported by Zhan et al. in septic AKI and the L-shaped association reported by He et al. in critically ill pediatric patients [13,23], the risk in the low FAR range was relatively flat in our study, with no phenomenon of increased mortality risk at very low FAR values. This discrepancy may be related to the clearance effect of CRRT and is also consistent with the observation by Amouzandeh et al. that in critical illness, albumin synthesis declines while fibrinogen synthesis can be maintained at normal levels [25]. The approximately linear positive association of UAR is consistent with the findings of Özgür et al. and Sargin Ertan et al. [15,16]. Subgroup analysis showed that the combined high-FAR/high-UAR indicator was significantly associated with mortality risk in most subgroups, with SOFA score (*p* for interaction = 0.019) and vasopressin use (*p* for interaction = 0.022) having significant modifying effects. Patients with more severe illness (SOFA > 12) or those receiving vasopressin had higher HRs (2.06 and 2.07, respectively). The HR for non-sepsis patients (2.06) was higher than that for sepsis patients (1.56). Taken together with the antioxidant function of albumin described by Belinskaia et al. [24], this suggests that oxidative stress injury may be more prominent in non-infectious AKI.

Our study has several limitations that must be acknowledged. First, its retrospective observational design precludes causal inference. Second, laboratory measurements were based on the first ICU values and did not account for dynamic changes during treatment. Nevertheless, in clinical practice, the first measurements upon ICU admission are routinely used for initial risk stratification and immediate treatment decisions, which supports the clinical relevance of our approach. However, due to irregular sampling intervals in the MIMIC-IV database, time-varying covariate analysis was not feasible. It is worth noting that dynamic changes in FAR and UAR may influence mortality risk estimates, and future prospective studies with serial sampling are needed to address this issue. Third, the optimal cut-off values for FAR and UAR were determined solely based on the MIMIC-IV cohort, which reflects the demographic characteristics and clinical protocols of a single medical center. Therefore, these specific thresholds may lack generalizability and should not be directly applied to other clinical settings without external validation. Readers are advised to use these cut-off values with caution. Fourth, although an international database was used, further validation through multicenter, prospective studies are still required to generalize our findings. Additionally, we did not adjust for pre-admission medications that might directly affect uric acid levels. The MIMIC-IV database does not provide complete and reliable records of out-hospital medication use before ICU admission, and in-hospital medications are time-varying variables, which limits their inclusion as baseline confounding factors. Therefore, unmeasured confounding factors related to medication use may have influenced the observed associations for UAR. Furthermore, we were unable to adjust for specific CRRT parameters, including prescribed dose, delivered dose, treatment duration, and modality, because these data were not consistently recorded in the MIMIC-IV database. Detailed nutritional intake data that directly affect albumin and other laboratory parameters were also unavailable. These unmeasured factors may confound the observed associations and should be addressed in future prospective studies.

## 5. Conclusions

In this retrospective cohort study of critically ill patients with acute kidney injury undergoing continuous renal replacement therapy, the combined presence of a high fibrinogen-to-albumin ratio and a high uric acid-to-albumin ratio was independently associated with elevated short- and long-term all-cause mortality. These readily obtainable composite markers may serve as adjunctive tools for risk stratification in this high-risk population.

## Figures and Tables

**Table 1:** Baseline characteristics of AKI patients undergoing CRRT stratified by 30-day survival status.

Variables	Total	Survival Group	Non-Survival Group	*p*-Value
	*n* = 901	*n* = 433	*n* = 468	
Age (year)	63.00 (52.00, 73.00)	61.00 (50.00, 71.00)	65.00 (53.00, 74.00)	<0.01
Gender, *n* (%)				0.17
Female	360 (39.96)	163 (37.6)	197 (42.09)	
Male	541 (60.04)	270 (62.36)	271 (57.91)	
Race, *n* (%)				<0.01
White	498 (55.27)	261 (60.28)	237 (50.64)	
Asian	21 (2.33)	10 (2.31)	11 (2.35)	
Black	73 (8.10)	42 (9.70)	31 (6.62)	
Others	309 (34.30)	120 (27.71)	189 (40.39)	
BMI	31.24 (26.83, 37.09)	31.48 (26.87, 37.56)	30.97 (26.81, 36.39)	0.39
APS III	82.00 (67.00, 99.00)	76.00 (63.00, 91.00)	88.00 (73.00, 104.00)	<0.01
SOFA score	12.00 (9.00, 14.00)	11.00 (8.00, 14.00)	12.00 (10.00, 15.00)	<0.01
**Laboratory parameters**				
Fibrinogen (g/L)	17.70 (14.20, 24.70)	17.00 (13.90, 22.70)	18.55 (14.60, 27.35)	<0.01
Uric acid (umol/L)	5.40 (3.80, 7.00)	5.20 (3.70, 6.90)	5.50 (3.98, 7.20)	0.052
Albumin (g/dL)	3.00 (2.50, 3.70)	3.10 (2.50, 4.40)	2.90 (2.40, 3.60)	<0.01
FAR	6.05 (4.48, 8.81)	5.52 (4.06, 7.71)	6.67 (4.73, 9.88)	<0.01
UAR	1.65 (1.17, 2.48)	1.50 (1.05, 2.26)	1.77 (1.27, 2.64)	<0.01
WBC (×10^9^/L)	13.70 (8.80, 19.90)	12.80 (8.20, 18.40)	15.00 (9.60, 21.20)	<0.01
Platelet (×10^9^/L)	140.00 (81.00, 225.00)	143.00 (85.00, 224.00)	139.00 (78.00, 226.00)	0.43
Hemoglobin (g/L)	9.80 (8.10, 11.80)	9.80 (8.30, 11.90)	9.70 (8.00, 11.80)	0.21
RDW (%)	15.80 (14.20, 18.40)	15.60 (14.10, 17.80)	15.90 (14.20, 18.80)	0.02
Lymphocyte (×10^9^/L)	0.94 (0.51, 1.56)	0.92 (0.51, 1.59)	0.95 (0.49, 1.52)	0.76
Neutrophil (×10^9^/L)	11.45 (7.30, 17.08)	10.76 (6.60, 16.31)	12.12 (8.12, 17.51)	<0.01
Monocyte (×10^9^/L)	0.82 (0.40, 1.38)	0.77 (0.35, 1.36)	0.86 (0.46, 1.39)	0.12
PCO2 (mmhg)	40.00 (34.00, 48.00)	40.00 (34.00, 48.00)	40.00 (34.00, 50.00)	0.53
PO2 (mmhg)	89.00 (55.00, 161.00)	91.00 (61.00, 184.00)	87.00 (52.00, 151.00)	0.05
AST (U/L)	120.00 (47.00, 496.00)	113.00 (43.00, 573.00)	125.00 (50.75, 439.50)	0.54
ALT (U/L)	58.00 (25.00, 257.00)	57.00 (25.00, 305.00)	58.00 (26.00, 212.25)	0.81
Creatinine (umol/L)	2.40 (1.60, 3.80)	2.50 (1.60, 4.00)	2.35 (1.50, 3.60)	0.12
BUN (mg/dL)	39.00 (23.00, 65.00)	40.00 (23.00, 63.00)	37.00 (23.75, 65.25)	0.85
Glucose (mmol/L)	140.00 (104.00, 201.00)	140.00 (107.00, 200.00)	141.00 (102.75, 202.25)	0.58
Sodium (mmol/L)	138.00 (133.00, 141.00)	138.00 (133.00, 141.00)	138.00 (133.00, 142.00)	0.52
Total calcium (mmol/L)	8.10 (7.30, 8.80)	8.10 (7.30, 8.90)	8.10 (7.40, 8.80)	0.72
Potassium (mmol/L)	4.60 (4.00, 5.30)	4.50 (4.00, 5.20)	4.60 (4.00, 5.30)	0.55
Chlorine (mmol/L)	102.00 (96.00, 107.00)	102.00 (97.00, 107.00)	101.00 (96.00, 107.00)	0.13
Magnesium (mmol/L)	2.10 (1.80, 2.50)	2.10 (1.80, 2.40)	2.20 (1.90, 2.50)	0.01
Anion gap (mmol/L)	19.00 (15.00, 23.00)	18.00 (15.00, 22.00)	19.00 (15.00, 24.00)	<0.01
**Therapies, *n* (%)**				
MV	729 (80.91)	342 (78.98)	387 (82.69)	0.16
TTE	34 (3.77)	21 (4.85)	13 (2.78)	0.10
Norepinephrine	504 (55.94)	214 (49.42)	290 (61.97)	<0.01
Dopamine	62 (6.88)	28 (6.47)	34 (7.27)	0.64
Adrenaline	151 (16.76)	56 (12.93)	95 (20.30)	<0.01
Vasopressin	315 (34.96)	120 (27.71)	195 (41.67)	<0.01
Dobutamine	43 (4.77)	17 (3.92)	26 (5.56)	0.25
Phenylephrine	220 (24.42)	110 (25.40)	110 (23.50)	0.51
**Comorbidities, *n* (%)**				
Hypertension	531 (58.94)	259 (59.82)	272 (58.12)	0.61
Diabetes	299 (33.19)	160 (36.95)	139 (29.70)	0.02
Cirrhosis	138 (15.32)	64 (14.78)	74 (15.81)	0.67
Chronic pulmonary disease	198 (21.98)	92 (21.25)	106 (22.65)	0.61
AMI	146 (16.20)	55 (12.70)	91 (19.44)	<0.01
Sepsis	511 (56.72)	218 (50.35)	293 (62.61)	<0.01
Oliguria	886 (98.34)	424 (97.92)	462 (98.72)	0.35
Heart Failure	305 (33.86)	141 (32.56)	164 (35.04)	0.43
Ischemic Stroke	53 (5.88)	24 (5.54)	29 (6.120)	0.68
Malignant tumor	150 (16.65)	76 (17.55)	74 (15.81)	0.48

Abbreviations: BMI: body mass index; APS III: acute physiology score III; SOFA: sequential organ failure assessment; FAR: fibrinogen-to-albumin ratio; UAR: uric acid-to-albumin ratio; WBC: white blood cell; RDW: red blood cell distribution width; PO2: partial pressure of oxygen; PCO2: partial pressure of carbon dioxide; AST: aspartate aminotransferase; ALT: alanine aminotransferase; BUN: blood urea nitrogen; MV: mechanical ventilation; TTE: transthoracic echocardiography; AMI: acute myocardial infarction.

**Table 2:** Cox proportional hazards model assessing all-cause mortality in patients.

Variables	Model 1		Model 2		Model 3	
	HR (95%CI)	*p*	HR (95%CI)	*p*	HR (95%CI)	*p*
FAR and UAR	30-day mortality					
1	1.00 (Reference)		1.00 (reference)		1.00 (reference)	
2	1.71 (1.15~2.54)	<0.01	1.74 (1.17~2.59)	<0.01	1.59 (1.06~2.37)	0.02
3	1.67 (1.22~2.29)	<0.01	1.65 (1.21~2.26)	<0.01	1.61 (1.17~2.22)	<0.01
4	2.55 (1.90~3.41)	<0.01	2.65 (1.98~3.55)	<0.01	2.17 (1.61~2.92)	<0.01
	360-day mortality					
1	1.00 (Reference)		1.00 (reference)		1.00 (reference)	
2	1.31 (0.94~1.82)	0.11	1.35 (0.97~1.87)	0.08	1.21 (0.87~1.70)	0.25
3	1.20 (0.93~1.54)	0.15	1.17 (0.91~1.51)	0.21	1.14 (0.88~1.47)	0.31
4	1.72 (1.37~2.17)	<0.01	1.78 (1.42~2.25)	<0.01	1.50 (1.18~1.90)	<0.01

Model 1: Crude. Model 2: Adjust: Age, gender, BMI. Model 3: Adjust: Age, gender, BMI, sepsis, diabetes, AMI, vasopressin, SOFA score, RDW, neutrophil, PO2, creatinine, magnesium, Anion gap. Abbreviations: BMI: body mass index; AMI: acute myocardial infarction; SOFA: sequential organ failure assessment; RDW: red blood cell distribution width; PO2: partial pressure of oxygen; HR: hazard ratio; CI: confidence interval. Low FAR/low UAR (1), high FAR/low UAR (2), low FAR/high UAR (3), high FAR/high UAR (4).

## Data Availability

The datasets used and analyzed during the current study are available from MIMIC-IV.

## Supplementary Materials

The following supporting information can be downloaded at: https://www.mdpi.com/article/10.3390/jcm15093271/s1, Figure S1. ROC curves for FAR, UAR, and their combination in predicting 30-day mortality. Figure S2. RCS analyses of the correlation between FAR, UAR, and the risk of 30-day all-cause mortality after excluding patients with cirrhosis.

## Abbreviations

The following abbreviations are used in this manuscript:

AKI	Acute Kidney Injury
CRRT	Continuous Renal Replacement Therapy
FAR	Fibrinogen-to-Albumin Ratio
UAR	Uric Acid-to-Albumin Ratio
MIMIC-IV	Medical Information Mart for Intensive Care-IV
ICU	Intensive Care Unit
ESRD	End-Stage Renal Disease
KDIGO	Kidney Disease: Improving Global Outcomes
APS III	Acute Physiology Score III
SOFA	Sequential Organ Failure Assessment
BMI	Body Mass Index
RDW	Red Cell Distribution Width
PCO2	Partial Pressure of Carbon Dioxide
PO2	Partial Pressure of Oxygen
AST	Aspartate Aminotransferase
ALT	Alanine Aminotransferase
BUN	Blood Urea Nitrogen
MV	Mechanical Ventilation
TTE	Transthoracic Echocardiography
AMI	Acute Myocardial Infarction
ICD-9/ICD-10	International Classification of Diseases, Ninth or Tenth Revision
IQR	Interquartile Range
HR	Hazard Ratio
CI	Confidence Interval
ROC	Receiver Operating Characteristic
RCS	Restricted Cubic Spline
STROBE	Strengthening the Reporting of Observational Studies in Epidemiology
MIT	Massachusetts Institute of Technology
