# Supplementary material for: Combined Association of the Fibrinogen-to-Albumin Ratio and the Uric Acid-to-Albumin Ratio with Mortality in Critically Ill Patients with Acute Kidney Injury Receiving Continuous Renal Replacement Therapy: A Retrospective Cohort Study
Source: J Clin Med. 2026 Apr 24;15(9):3271. doi: 10.3390/jcm15093271 (PMC13163587; doi:10.3390/jcm15093271)
Supplement: Supplementary file 1 [file jcm-15-03271-s001.zip › jcm-4179732-supplementary.pdf]

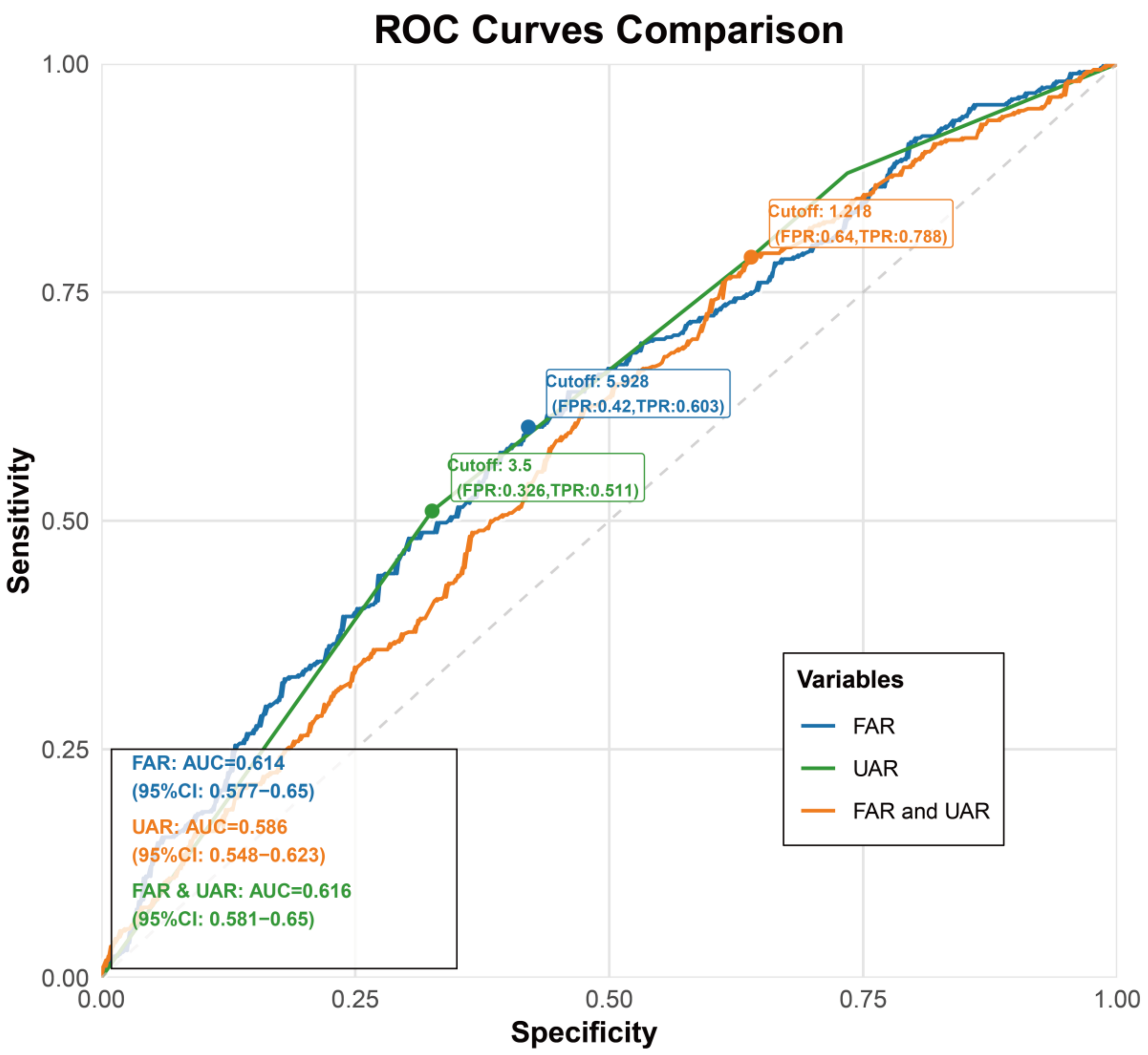

Supplementary Figure 1. Receiver operating characteristic (ROC) curves for the fibrinogen-to-albumin ratio (FAR), the uric acid-to-albumin ratio (UAR), and their combination for discriminating 30-day all-cause mortality. The area under the curve (AUC) was 0.614 (95% confidence interval [CI]: 0.577–0.650) for FAR, 0.586 (95% CI: 0.548–0.623) for UAR, and 0.616 (95% CI: 0.581–0.650) for the combination. Optimal cut-off values, determined by the Youden index, are indicated on the respective curves.

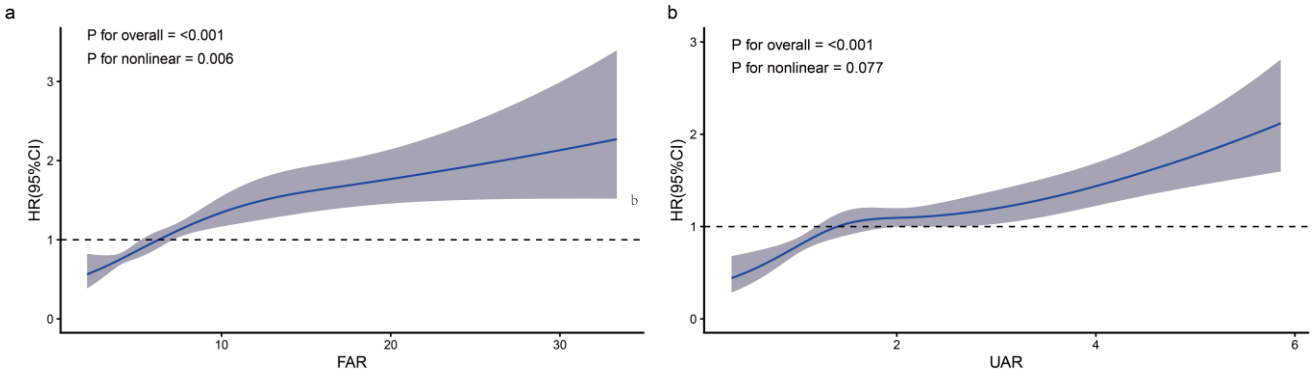

Supplementary Figure S2. Restricted cubic spline (RCS) analysis of the dose–response relationship between (a) the fibrinogen-to-albumin ratio (FAR) and (b) the uric acid-to-albumin ratio (UAR) with 30-day all-cause mortality after exclusion of patients with cirrhosis (n = 763). Solid lines represent the estimated hazard ratio (HR), and shaded bands indicate 95% confidence intervals (CI). The reference values (HR = 1.0) were set as the medians of FAR and UAR.
